# Identification of cuproptosis hub genes contributing to the immune microenvironment in ulcerative colitis using bioinformatic analysis and experimental verification

**DOI:** 10.3389/fimmu.2023.1113385

**Published:** 2023-03-07

**Authors:** Cejun Yang, Wendi Wang, Sang Li, Zhengkang Qiao, Xiaoqian Ma, Min Yang, Juan Zhang, Lu Cao, Shanhu Yao, Zhe Yang, Wei Wang

**Affiliations:** ^1^The Institute for Cell Transplantation and Gene Therapy, The Third Xiangya Hospital of Central South University, Changsha, China; ^2^Department of Radiology, The Third Xiangya Hospital of Central South University, Changsha, China; ^3^College of Life Science, Liaoning University, Shenyang, China; ^4^Department of Research, Engineering and Technology Research Center for Xenotransplantation of Human Province, Changsha, China

**Keywords:** ulcerative colitis, colorectal cancer, cuproptosis, immune microenvironment, bioinformatics analysis, experimental verification

## Abstract

**Instruction:**

Ulcerative colitis (UC) can cause a variety of immune-mediated intestinal dysfunctions and is a significant model of inflammatory bowel disease (IBD). Colorectal cancer (CRC) mostly occurs in patients with ulcerative colitis. Cuproptosis is a type of procedural death that is associated with different types of diseases to various degrees.

**Methods:**

We used a combination of bioinformatic prediction and experimental verification to study the correlation between copper poisoning and UC. We used the Gene Expression Omnibus database to obtain disease gene expression data and then identified relevant genes involved in various expression levels in normal and UC samples. The Kyoto Encyclopedia of Genes and Genomes pathway analysis was performed to cluster the genes that are highly responsible and find the central interaction in gene crosstalk. Notably, *DLD*, *DLAT*, and *PDHA1* were present in high-scoring PPI networks. In addition, hub gene expression information in UC tissues was integrated to estimate the relationship between UC copper poisoning and the immune environment.

**Results:**

In our study, the expression of *DLD*, *DLAT*, and *PDHA1* in UC tissues was lower than that in normal tissues. The key genes associated with cuproptosis have therapeutic effects on immune infiltration. We verified the expression of *DLD*, *DLAT*, and *PDHA1* using real-time quantitative polymerase chain reaction in mouse models of UC induced by DSS.

**Discussion:**

Notably, this study clearly indicates that bioinformatic analysis performed to verify the experimental methods provides evidence that cuproptosis is associated with UC. This finding suggests that immune cell infiltration in UC patients is associated with cuproptosis. The key genes associated with cuproptosis can be helpful for discovering the molecular mechanism of UC, thus facilitating the improvement of UC treatment and preventing the associated CRC.

## 1 Introduction

Ulcerative colitis (UC) is a chronic inflammatory disease of the intestine that significantly influences human life and health. According to population-based studies, people with UC have a higher chance of developing colorectal cancer (CRC) than those without UC. Notably, CRC is a well-known and serious consequence that frequently affects patients with UC (1). Currently, the underlying causes and mechanisms of UC are not completely understood. The clinical features of this condition include weight loss, vomiting, fecal urgency, and other related symptoms (2). Chronic inflammatory stimulation of the colon significantly increases the incidence of CRC in UC patients, and colitis-related malignancy is the main cause of death in UC patients. Despite extensive investigation, the reasons and mechanisms of this change remain unknown (3, 4).

Cuproptosis plays a significant role in the occurrence and development of cancer and has been widely studied as a new mechanism. It is characterized by the presence of copper ions and by mitochondrial respiration. However, the comprehensive relationship between cuproptosis, UC, and CRC remains unclear. Notably, copper can accelerate cell proliferation by activating the RAS signaling cascade (5). Furthermore, copper is sensitive to many angiogenic factors that accelerate tumor growth and transfer (6).

Bioinformatics plays a significant role in the early diagnosis, effective treatment, and prediction of clinical challenges in key genes of patients (7). This innovative approach has been widely used to study various cancer types (8) and in the recognition of new biomarkers for several non-neoplastic diseases (9). Therefore, we identified three genes that are associated with cuproptosis in an earlier study (10, 11). The RNA sequencing data of UC patients and healthy control samples were downloaded from the GEO database, and bioinformatic analysis was performed. In addition, we constructed a mouse model of UC. We also performed a differential analysis of their expression profiles. All three cuproptosis-related genes were found to be differentially expressed. Currently, no reports on the biological role of cuproptosis in UC or in the immune microenvironment are available. We discussed the close association between cuproptosis and UC. In summary, this study reveals that cuproptosis has a significant influence on UC pathogenesis, and provides a new strategy to treat UC and UC-related CRC.

## 2 Materials and methods

### 2.1 Enrichment analysis of differential genes

The intersection of differential genes between UC and normal samples analyzed from the GSE38713, GSE87473, and GSE92415 datasets yielded 1,678 genes that were downregulated in UC and 1,605 genes upregulated in UC. Metascape is an analytical website integrated with functional analyses of GO and KEGG (12). We set the screening conditions as Min overlay = 3 and Min enrichment = 1.5 and used Metascape to predict the enrichment of differential gene intersection. Statistical significance was set at *p*-value <0.01 (13). We used the R package clusterProfiler to predict the different degrees of enrichment at various levels of expressed genes in the data.

### 2.2 Acquisition of data

The GEO database can be used as a common functional genome warehouse for high-throughput gene expression data, chips, and microarrays to qualitatively study the expression of disease-related genes (https://www.ncbi.nlm.nih.gov/geo/). We obtained the GSE38713, GSE16879, GSE87473, and GSE92415 datasets from the GEO database (14–16). The GSE38713 dataset, containing colon biopsy samples from 13 non-inflammatory controls and 30 UC patients, was conducted using Affymetrix Human Genome U133 Plus 2.0 Array. The basic analysis is contained in the manuscript published in the *Gut* journal with PMID 23135761. The GSE87473 (PMID: 29401083), containing colon mucosal biopsies from 21 healthy participants and 106 UC patients, was conducted using Affymetrix HT HG-U133+ PM Array Plate. The GSE92415 dataset, containing colonic mucosal biopsy samples from 21 healthy participants and 53 UC patients, was conducted using Affymetrix HT HG-U133+ PM Array Plate. The basic analysis is contained in the manuscript published in *Gastroenterology* with PMID 23735746. The GSE16879 dataset contains 48 UC samples and 12 normal samples. Total RNA was isolated from intestinal mucosal biopsies, labeled, and hybridized to Affymetrix Human Genome U133 Plus 2.0 Arrays. The basic analysis is contained in the manuscript published in *PLoS One* with PMID 19956723. The GSE16879 is a mucosal expression profile microarray in patients with inflammatory bowel disease before and after the first infliximab treatment (**Table S3**) (17). Our annotation information for the reference platform data converted the probe IDs into the corresponding gene symbols. We obtained the raw “CEL” files for the two datasets, called the AffyPLM package in R, and normalized them using the RMA algorithm (18). On differential expression analysis, *p*-values <0.05 were considered statistically significant.

### 2.3 PPI network development and module research

STRING (version 11.5) was used to build a protein–protein interaction (PPI) network for the differential genes (19). Cytoscape (Version 3.9.1) was used for visual analysis of the interaction network between proteins (20). Molecular Complex Detection (MCODE) (Version 2.0) uses the inherent relationship between proteins in the network to find gene clusters and build a foundation for subsequent analysis (21). We used Cytoscape to read the PPI network files and then used the MCODE plugin to identify the key modules in the PPI network.

### 2.4 Sample classification

The ConsensusClusterPlus package in R was used to classify patients with quality differences in cuproptosis-related genes. The *k* value was selected and determined by defining the inflection point of the sum of errors.

### 2.5 Screening differential genes in cuproptosis-related gene clustering

Differential genes of cluster patterns were screened using the limma package. The expression profiles of the cluster patterns were obtained. The parameters were set at *p <*0.05.

### 2.6 Analysis of immune infiltration and efficacy differences

The degree of each sample was obtained using the single-sample gene set enrichment analysis (ssGSEA) algorithm, and after downloading the gmt format gene-set data required for the analysis, the samples in GSE87473 were immunoscored using the R package GSVA, containing a total of 23 immune-related scores. The differential expression of immune checkpoint genes (*PD-L1*, *IDO1*, *HAVCR2*, *PDCD1LG2*, *CD86*, *ICOS*, *TNFRSF9*, and *CTLA4*) and their response to anti-TNF therapy were analyzed (22–24).

### 2.7 Daily observation and sample collection

While the feeding and drinking habits of each mouse were documented, a daily and routine inspection of the appearance of each mouse (vitality and hair) was also performed. Body weight, stool consistency, and degree of intestinal bleeding were recorded, and the disease activity index (25) was calculated (**Table S14**). The colon was promptly removed when mice were sacrificed using the cervical dislocation technique on the 7^th^ day, and colon lengths were obtained. A portion of the colon samples was preserved at −80°C for further studies, while the remainder of the colon tissue was sliced (1 cm) and fixed in paraformaldehyde (4%) for histological testing (26).

### 2.8 Histopathological assessment

The tissue sample was embedded in paraffin after fixing with paraformaldehyde (4%). The tissue was cut to a thickness of 5 mm and dyed with fuel. The slices were then examined under a light microscope for blind analysis. **Table S15** displays the histological scores (26).

### 2.9 Real-time quantitative polymerase chain reaction

Freshly frozen tissues were maintained at −80°C. First, the RNA associated with the colon tissue proteins was isolated using TRIzol reagent and a Thermo reverse transcription kit. Second, using an ABI7500 fluorescence quantitative PCR instrument and PerfectStart^®^ Green qPCR SuperMix amplification reagent, PCR amplification and detection were performed with actin as the internal control. The expression levels were examined using the 2^−ΔΔCt^ approach. The primer sequences were as follows: DLAT-F: GAGGTGCTGTTGGTACGGAA, DLAT-R: ACGAGTTTGCTTCGGGAACT; DLD-F: AGTCGTGTGTACCGCTCCTT, DLD-R: CACTGTCACGTCAGCCTCAA; and PDHA1-F: GGATGGAGCTAAAGGCGGAT, PDHA1-R: TCCGTAGGGTTTAT GCCAGC.

### 2.10 Western blot analysis

Cells were lysed using RIPA buffer (Tris 20 mM, NaCl 150 mM, KCl 20 mM, MgCl_2_ 1.5 mM, glycerol 10%, Triton X-100 1%, pH 7.5), and the protein concentration was measured by the BCA Protein Assay Kit (C503021; Sangon Biotech, Shanghai, China). Equal amounts of proteins were separated by 10%–12% sodium dodecyl sulfate-polyacrylamide gel electrophoresis and were transferred onto polyvinylidene fluoride membranes, and the target proteins were finally detected using standard Western blotting protocols and visualized using the Super Signal West Pico Plus Luminol/Enhancer Solution (UC279012; Thermo, Waltham, USA). β-Actin was used as the loading control. The primary antibodies used were listed as follows unless otherwise specified: anti-β-actin antibody (AC038; ABclonal, Wuhan, China), anti-DLD antibody (A5220; ABclonal), anti-PDHA1 antibody (A13687; ABclonal), anti-DLAT antibody (A8814; ABclonal). The secondary antibodies used were as follows unless otherwise specified: horseradish peroxidase (HRP)-conjugated goat anti-rabbit IgG (H + L) (AS014; ABclonal).

### 2.11 Statistical analysis

Most analyses used the R software, partly using the GraphPad Prism 8 software and the Sangerbox (27) website. In all statistical analyses, *p <*0.05 was significantly different. The *p*-value of the differential gene selection has been corrected (https://www.biocon.5octor.org/, https://www.graphpad.com/, http://sangerbox\2/login.html).

## 3 Results

### 3.1 Screening for differentially expressed genes in ulcerative colitis

**Figure 1:**
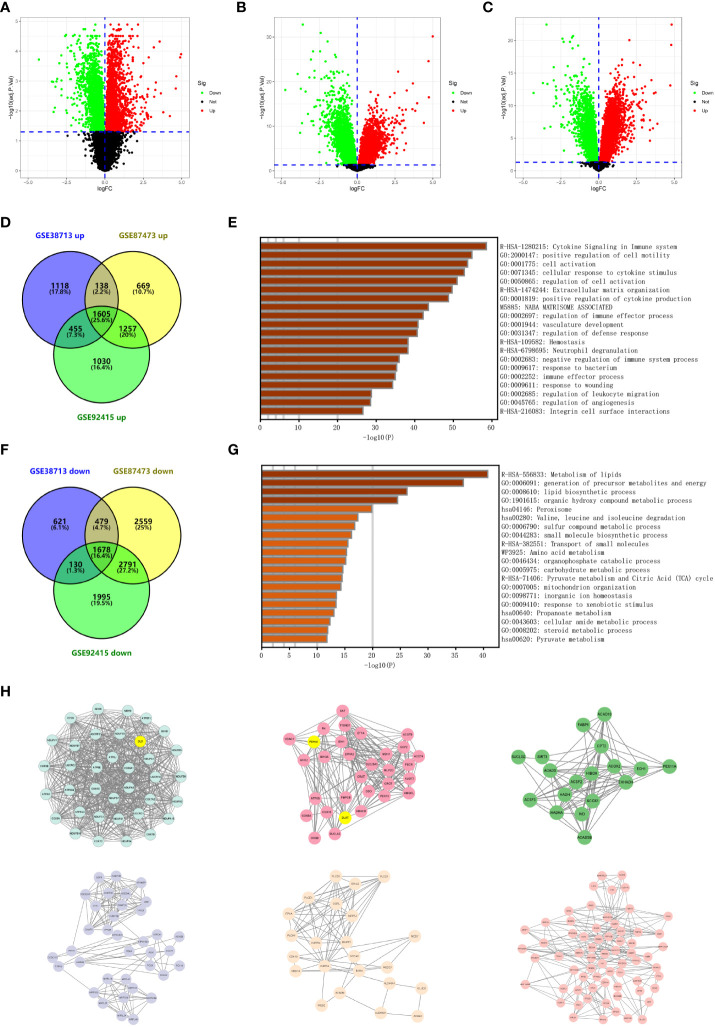
Identification of differentially expressed genes in ulcerative colitis. **(A)** Volcano plot depicting the differentially expressed genes of microarray GSE38713. Red data points represent upregulated genes and green data points represent downregulated genes. Genes without any significant differences are in black. **(B)** Volcano plot depicting the differentially expressed genes of microarray GSE87473. Red data points represent upregulated genes and green data points represent downregulated genes. Genes without any significant differences are in black. **(C)** Volcano plot depicting the differentially expressed genes of microarray GSE92415. Red data points represent upregulated genes and green data points represent downregulated genes. Genes without any significant differences are in black. **(D)** The Venn diagram depicts the overlap of upregulated genes between the three datasets retrieved from the GEO database. **(E)** Functional enrichment analysis of overlapping upregulated genes. The bar graph depicts the top 20 results of the upregulated gene enrichment analysis; *p*-values are differentiated by shades of color. **(F)** The Venn diagram depicts the overlap of downregulated genes between the three datasets retrieved from the GEO database. **(G)** Functional enrichment analysis of overlapping downregulated genes. The bar graph depicts the top 20 results of downregulated gene enrichment analysis; *p*-values are differentiated by shades of color. **(H)** The key subnetworks in the PPI network are calculated using the MCODE algorithm in Cytoscape software, and the subnetworks with the highest weights and the sixth ranked subnetworks are presented from the top left to the bottom right, respectively.

We performed a differential analysis of the expression between the normal and UC tissue samples in three datasets (GSE38713, GSE87473, and GSE92415) using the limma package (**Figures 1A–C** and **Table S1**). The upregulated and downregulated differentially expressed genes intersected in GSE38713, GSE87473, and GSE92415 (**Table S2**). We finally obtained 1,605 upregulated genes and 1,678 downregulated genes (**Figures 1D, F**). We used Metascape to predict the enrichment of the intersection of the differential genes (**Figures 1E, G**). Most of the upregulated genes were enriched in cytokine signaling in the immune system and positive regulation of cell motility, and the downregulated genes were enriched in the metabolism of lipids, generation of precursor metabolites, and energy and lipid biosynthetic processes.

The most critical modules in the PPI network were recognized using the MCODE plugin in Cytoscape. This article indicates the top six subnetworks in the networks constructed by the downregulated genes. The weight of these networks from left to right is 33.314, 14.067, 10.471, 8.227, 6.909, 6.727, 6.250, 6.000, 5.000, and 4.889 (**Figure 1H** and **Figure S1**). We then identified and marked three cuproptosis-associated genes in the top two ranked networks and analyzed them.

### 3.2 Expression levels of cuproptosis-associated genes in ulcerative colitis

**Figure 2:**
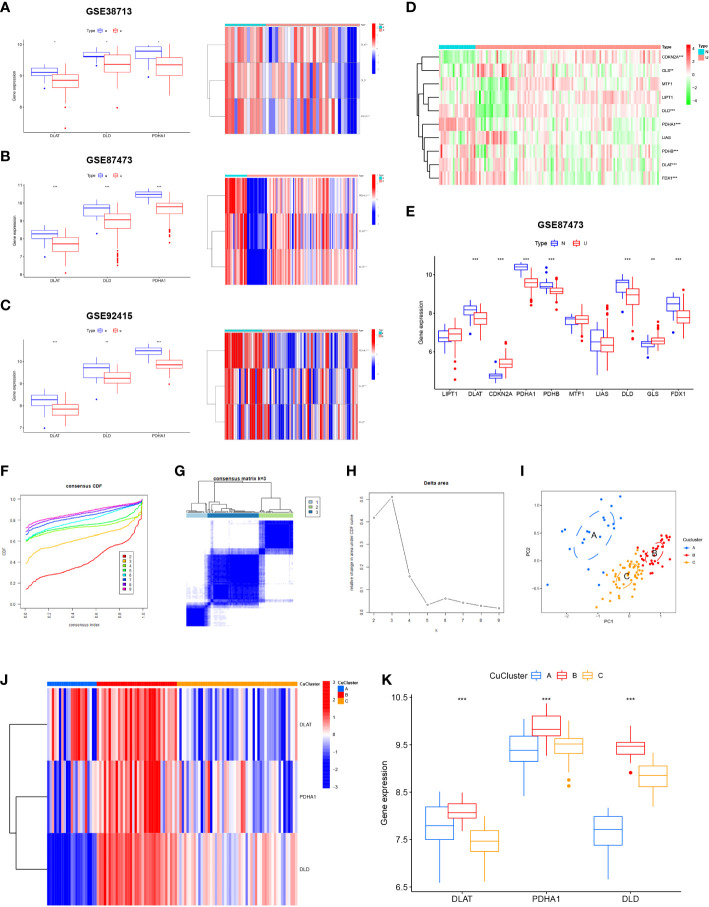
Expression of cuproptosis-related genes in ulcerative colitis (UC) patients. **(A)** Expression of three cuproptosis-related genes in GSE38713. **(B)** Expression of three cuproptosis-related genes in GSE92415. **(C)** Expression of three cuproptosis-related genes in GSE87473. **(D)** Heat map indicated the expression of 10 cuproptosis-related genes in normal tissues and UC patients. **(E)** Box line plot depicted the expression of 10 cuproptosis-related genes in normal tissues and UC patients. (**F–H**) Three subgroups were identified based on the expression of three cuproptosis-related genes using unsupervised consistent clustering. **(I)** Visualization of clustering results using the PCA method. **(J, K)** Evaluation of the expression of three cuproptosis-related genes in different cuproptosis subtypes. N, normal, normal tissues; U: UC, UC patients; CuClusterA, cuproptosis-ClusterA; CuClusterB, cuproptosis-ClusterB; CuClusterC, cuproptosis-ClusterC. The asterisk represents the statistical *p*-value (*p < 0.05; **p < 0.01; ***p < 0.001).

We analyzed the expression levels of the three cuproptosis-associated genes mentioned above in GSE38713, GSE92415, and GSE87473 (**Figures 2A-C**) based on the cuproptosis-associated genes studied by Tsvetkov et al. (26). To determine whether these cuproptosis-related genes affected disease progression in UC patients, we chose GSE87473 with a large sample size to assess the expression levels of cuproptosis-associated genes in UC and normal samples. The results indicate that seven genes (*DLAT*, *CDKN2A*, *PDHA1*, *PDHB*, *DLD*, *GLS*, and *FDX1*) were differentially expressed. We found high expression levels of *GLS* and *CDKN2A* in UC tissues (*p* < 0.01) and high expression levels of the other five genes in the normal tissues (*p* < 0.001) (**Figures 2D, E**). The results revealed that cuproptosis-associated genes indicated highly heterogeneous expression in normal and UC patients, suggesting that the expression of different cuproptosis-associated genes can affect disease progression in UC patients. Among them, 10 cuproptosis-associated gene expression profiles are presented in the Supplementary Material (**Table S3**). By analyzing the expression data from multiple ulcerative colitis, we found three cuproptosis-associated genes in the core subnetwork of the PPI network (**Figures S2A–F**). We finally selected the three hub genes *DLD*, *PDHA1*, and *DLAT* for the follow-up studies.

### 3.3 Consensus clustering of hub genes associated with cuproptosis in ulcerative colitis

Using the ConsensusClusterPlus package in R, the cuproptosis pattern was classified according to the expression levels of three cuproptosis-associated genes and identified as *k* = 3 (**Figures 2F–H**). Finally, we identified three different modification patterns through unsupervised clustering: 21 cases of CuClusterA, 34 cases of CuClusterB, and 51 cases of CuClusterC (**Table S4**). Furthermore, PCA indicated that the UC samples could be completely distinguished (**Figure 2I**). After unsupervised clustering, we studied cuproptosis-related genes in different CuClusters (**Figures 2J, K**). The strongest expression of cuproptosis-associated genes was found in CuClusterB (*p* < 0.001).

### 3.4 Cuproptosis patterns regulated the mucosal immune microenvironment in ulcerative colitis

**Figure 3:**
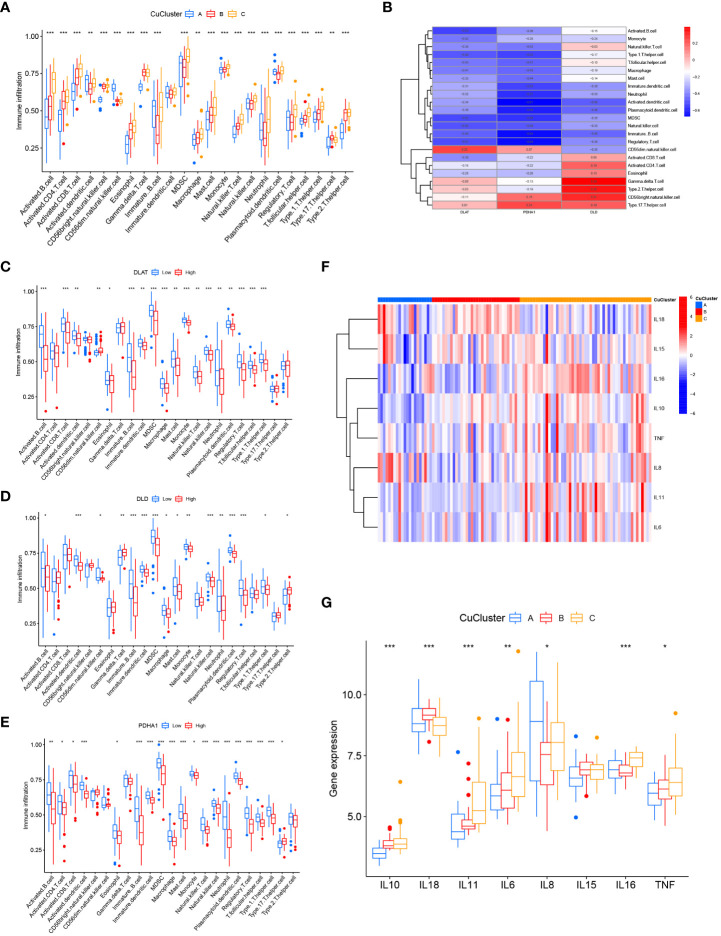
Consensus clustering of hub genes associated with cuproptosis in ulcerative colitis patients. **(A)** Differences in immune cell infiltration among CuClusterA, CuClusterB, and CuClusterC. **(B)** Correlation of three cuproptosis-related genes with mucosal immune cells. **(C–E)** Correlation between high and low expression of *DLAT* **(C)**, *DLD* **(D)**, *PDHA1* **(E)**, and mucosal immune cells. **(F, G)** Differences in inflammatory factors among CuClusterA, CuClusterB, and CuClusterC. CuClusterA, cuproptosis-ClusterA; CuClusterB, cuproptosis-ClusterB; CuClusterC, cuproptosis-ClusterC. The asterisk represents the statistical *p*-values (*p < 0.05; **p < 0.01; ***p < 0.001).

Next, we compared the differences in immune cell infiltration among the three clusters using ssGSEA analysis. The results indicated that multiple immune cells, except dendritic cells, were significantly enriched in the three CuClusters (**Figure 3A** and **Table S5**). Correlations between individual CuClusters and immune infiltration were analyzed using the online website Sangerbox (**Figure S3**). We then explored the association between these three cuproptosis-associated genes and immune infiltration (**Figure 3B**). *DLAT* expression positively correlated with CD56 dim natural killer cells (**Figure 3C**; *p* < 0.001). *DLD* expression positively correlated with activated CD8T cells, activated CD4T cells, eosinophils, γ-δ T cells, type 2 T helper cells, CD56 bright natural killer cells, and type 17 T helper cells. However, the correlation was only significant for γ-δT cells (*p* < 0.01) and type 2 T helper cells (**Figure 3D**; *p* < 0.05). *PDHA1* expression positively correlated with CD56 dim natural killer cells, CD56 bright natural killer cells, and type 17 T helper cells and significantly correlated with type 17 T helper cells (**Figure 3E**; *p* < 0.05). Moreover, we found that the three CuClusters, except IL15, were significantly differentially expressed in inflammatory factors (**Figures 3F, G**). Overall, cuproptosis-associated genes can affect the mucosal immune microenvironment of UC patients.

### 3.5 Biological characterization and consensus clustering analysis of differential genes

**Figure 4:**
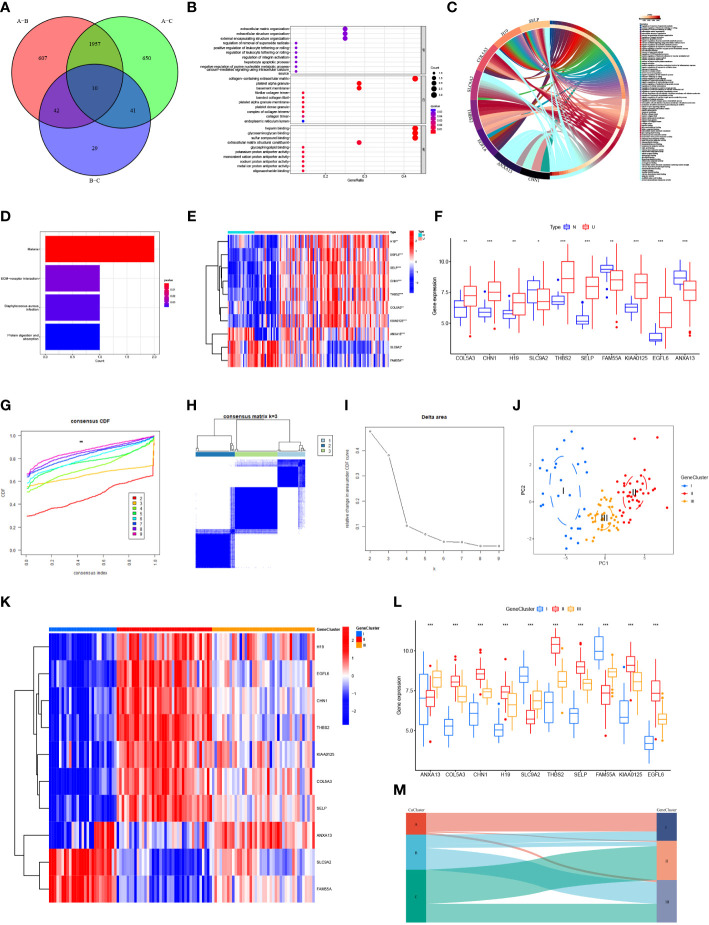
Biological characterization and consensus clustering analysis of differential genes. **(A)** Venn diagram of differentially expressed genes in three CuClusters. **(B)** Gene ontology (GO) enrichment analysis of differential genes: biological process (BP), cellular component (28), and molecular function (MF). **(C)** Visualization of differential gene enrichment using the Sangerbox website. **(D)** Kyoto Gene and Genome Encyclopedia (KEGG) pathway enrichment analysis of differential genes. **(E, F)** Expression of differentially expressed genes in normal tissues and in UC patients. (**G–I**) Clustering of samples based on differential gene expression using unsupervised consistent clustering to identify three subgroups. **(J)** Visualization of clustering results using the PCA method. **(K, L)** Expression of differential genes in different GeneClusters. **(M)** Alluvial diagram depicting the changes of CuClusters and GeneClusters. N, normal, normal tissues; U, UC, UC patients; CuClusterA, cuproptosis-ClusterA; CuClusterB, cuproptosis-ClusterB; CuClusterC, cuproptosis-ClusterC. The asterisk represents the statistical *p*-value (*p < 0.05; **p < 0.01; ***p < 0.001).

The number of differentially expressed genes in the three CuClusters was analyzed using the limma package, with a threshold of |logFC| >1 and *p <*0.05. CuClusterA–CuClusterB screened 2,616 differentially expressed genes (**Table S6**). CuClusterA–CuClusterC screened 2,658 differentially expressed genes (**Table S7**). CuClusterB–CuClusterC screened 122 differentially expressed genes (**Table S8**). Subsequently, the differential genes between the three CuClusters were intersected, and 10 differential genes were identified (**Figure 4A**). The pathway and enrichment information of the 10 genes were analyzed using the clusterProfiler R package, and two of the genes were not enriched for annotation (**Figure 4B** and **Table S9**). These genes were significantly enriched in extracellular matrix organization, extracellular structure organization, and external encapsulating structure organization (*p* < 0.05). In addition, these genes were also enriched in the collagen-containing extracellular matrix, platelet alpha granules, and basement membrane (*p* < 0.001). Among the cellular components, these genes were significantly enriched in heparin binding, glycosaminoglycan binding, and sulfur compound binding (*p* < 0.001). Differential genes were uploaded to the Sangerbox website for enrichment analysis (**Figure 4C**). Furthermore, enrichment in the KEGG pathway was observed for malaria, ECM receptor interaction, *Staphylococcus aureus* infection, and protein digestion and absorption (**Figure 4D** and **Table S10**; *p* < 0.05). After analyzing the expression levels of these 10 genes in GSE87473, we found that the expression of *COLA5A3*, *CHN1*, *H19*, *THBS2*, *SELP*, *KIAA0125*, and *EGFL6* was significantly upregulated in UC patients (**Figure 4E**; *p* < 0.05). The expression levels of *SLC9A2*, *FAM55A*, and *ANXA13* were significantly downregulated (**Figure 4F**; *p* < 0.05).

We classified UC patients into different genomic subtypes according to the differential expression levels of 10 genes associated with the cuproptosis phenotype. GeneClusters were well-distinguished (**Figures 4G–J** and **Table S11**). We found that these 10 genes were significantly different in GeneClusters (*p* < 0.05), with high expression of *SLC9A2* and *FAM55A* in GeneCluster I and *H19*, *EGFL6*, *CHN1*, *THBS2*, *KIAA0125*, *COL5A3*, and *SELP* in GeneCluster II. The expression of *ANXA13* was high in GeneCluster III (**Figures 4K, L**). Analysis of the results indicated that these 10 genes had the highest expression levels in GeneCluster II. On analyzing the relationship between CuClusters and GeneClusters using Sangerbox, we found that nearly all CuClusterA patients were from GeneCluster I and nearly all GeneCluster II patients were from CuClusterC (**Figure 4M**). These 10 genes have a strong association with cuproptosis-associated genes, suggesting that cuproptosis has a potential role in UC treatment.

### 3.6 Cuproptosis phenotype-associated hub genes affect immune infiltration and treatment response

**Figure 5:**
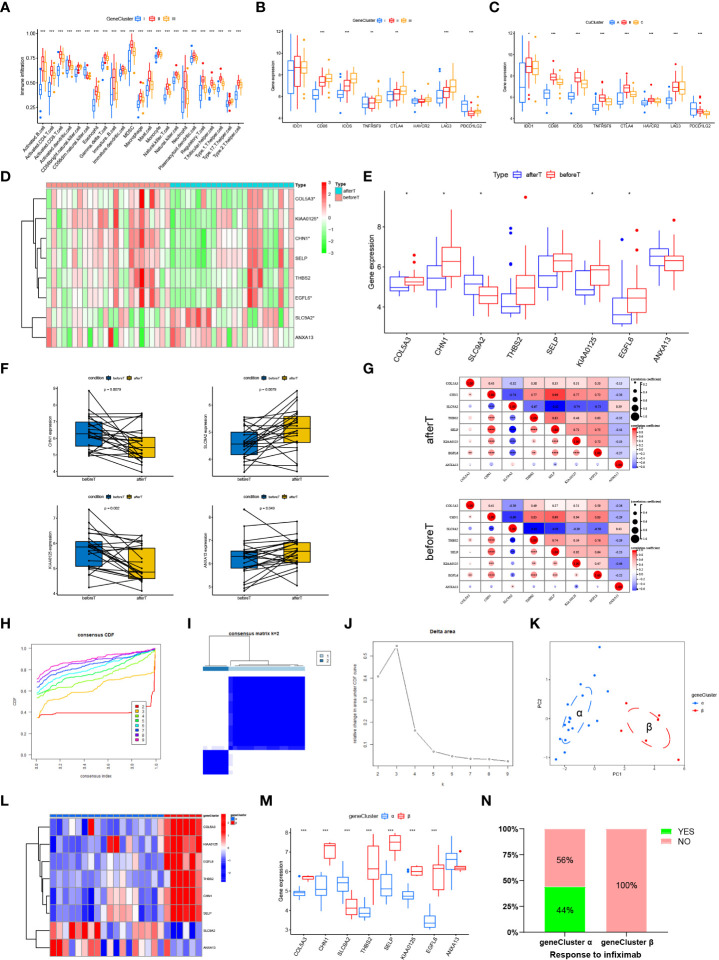
Cuproptosis phenotype-associated hub genes affect immune infiltration and treatment response. **(A)** Levels of immune cell infiltration in different GeneClusters. **(B, C)** Differential expression of eight important immune checkpoints in different CuClusters **(B)** and GeneClusters **(C)**. **(D, E)** The expression of eight hub genes in responders before and after the first infliximab treatment. **(F)** The expression of a single gene in responders before and after the first infliximab treatment. **(G)** Correlations between eight hub genes were analyzed in responders before and after the first infliximab treatment. **(H–J)** Clustering of samples based on cuproptosis phenotype-associated hub gene expression using unsupervised consistency clustering to identify three subgroups. **(K)** Visualization of clustering results using the PCA method. **(L, M)** Expression of cuproptosis phenotype-related hub genes in GeneClusters. **(N)** Treatment response of infliximab in different GeneClusters. N, normal, normal tissues; U, UC, UC patients; CuClusterA, cuproptosis-ClusterA; CuClusterB, cuproptosis-ClusterB; CuClusterC, cuproptosis-ClusterC. Before T represents responders before the first infliximab treatment. After T represents responders after the first infliximab treatment. The asterisk represents the statistical *p*-value (*p < 0.05; **p < 0.01; ***p < 0.001).

To explore the impact of cuproptosis phenotype-associated hub genes in UC immune infiltration, we evaluated the immune infiltration relationship between GeneCluster I, GeneCluster II, and GeneCluster III. The results indicated that CD56 dim natural killer cells were more abundant in GeneCluster I. Type 17 helper T cells were more abundant in GeneCluster III. Other immune cells were more abundant in GeneCluster II (**Figure 5A**, *p* < 0.05). Correlations between individual GeneClusters and immune infiltration were analyzed using Sangerbox (**Figure S4**). Notably, the expression of eight important immune checkpoints significantly differed between the CuClusters and GeneClusters (**Figures 5B, C**). We found that *CD86*, *ICOS*, *TNFRSF9*, *CTLA4*, and *LAG3* were expressed at their highest levels in CuClusterC (*p* < 0.05), and *PDCD1LG2* was the most highly expressed gene in CuClusterA (*p* < 0.05). We also found that, except for *PDCD1LG2*, the other seven immune monitoring sites exhibited the highest expression in GeneCluster II (*p* < 0.05), suggesting that the hub genes associated with the cuproptosis phenotype can affect immunotherapy.

The current treatment for patients with refractory and severe UC is infliximab, usually in combination with steroids, which was approved after clinical trials on these patients. Studies have indicated that infliximab treatment improves clinical presentation and induces mucosal healing in UC patients (29–32). Therefore, we further validated the expression levels of the 10 cuproptosis phenotype-associated hub genes in GSE16879. However, the expression results for only eight hub genes were available in GSE16879. The expression levels of these eight hub genes were analyzed in responders before the first infliximab treatment and after the first infliximab treatment (**Figures 5D, E** and **Table S12**). Furthermore, we analyzed the expression of a single gene in responders before and after the first infliximab treatment (**Figure 5F** and **Figure S5**). Correlations between eight hub genes were analyzed in responders before and after the first infliximab treatment (**Figure 5G**). The results indicated that the correlation between *KIAA0125* and *SLC9A2* and the correlation between *ANXA13* and *SELP* were both significantly enhanced, while the correlation between *ANXA13* and *KIAA0125* and the correlation between *THBS2* and *COLA53* were both significantly reduced after the first infliximab treatment compared with that before the first infliximab treatment. This suggests that the altered correlation between the genes is due to drug sensitivity. Based on the different expression levels of the eight hub genes, we performed a cluster analysis using the ConsensusClusterPlus R package, *k* = 2 (**Figures 5H–J** and **Table S13**). Notably, PCA indicated that the UC samples could be completely distinguished (**Figure 5K**). Furthermore, infliximab treatment was significantly different between the two gene clusters (**Figures 5L, M**). The hub genes (*COLA53*, *CHN1*, *THBS2*, *SELP*, *KIAA0125*, *EGFL6*) were significantly highly expressed in gene cluster α (*p* < 0.001), whereas *SLC9A2* was more expressed in cluster β than in gene cluster α (*p* < 0.05). *ANXA13* was not significantly differentially expressed in clusters α and β. Cluster α responded better to infliximab compared with cluster β (**Figure 5N**), suggesting that cuproptosis-associated hub gene expression can have implications in infliximab treatment.

In summary, the results suggested that high expression levels of some cuproptosis phenotype-associated hub genes in UC patients are sensitive to infliximab treatment. Analysis of the expression levels of cuproptosis phenotype-associated hub genes can facilitate the selection of clinical dosing.

### 3.7 Experimental verification in ulcerative colitis mouse model

**Figure 6:**
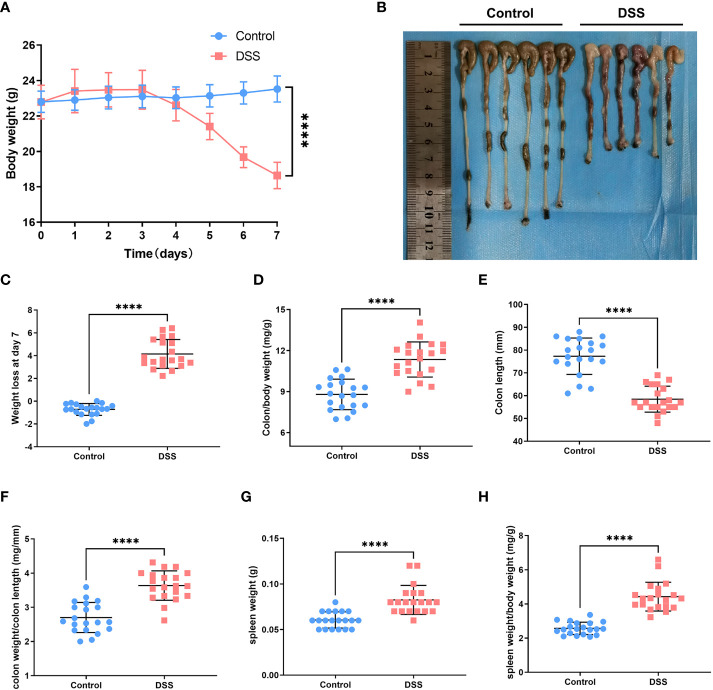
Experimental verification in the UC mouse model. **(A)** Changes in body weight of mice in the normal and dextran sulfate sodium (DSS) groups. **(B)** Colon length in the control group *versus* that in the DSS group. **(C)** DSS-treated mice had significant loss of body weight compared with control mice. **(D)** After acute DSS administration, the colon/body weight ratio was significantly higher in DSS-treated mice than in control mice. **(E)** Colon length decreased in DSS-treated mice compared with that in control mice. **(F)** Colon weight/length ratio increased after acute administration of DSS. **(G)** Spleen weight of mice in the DSS group was substantially higher than that of mice in the control group. **(H)** Spleen weight/body weight ratio increased after acute administration of DSS (****p < 0.0001).

**Figure 7:**
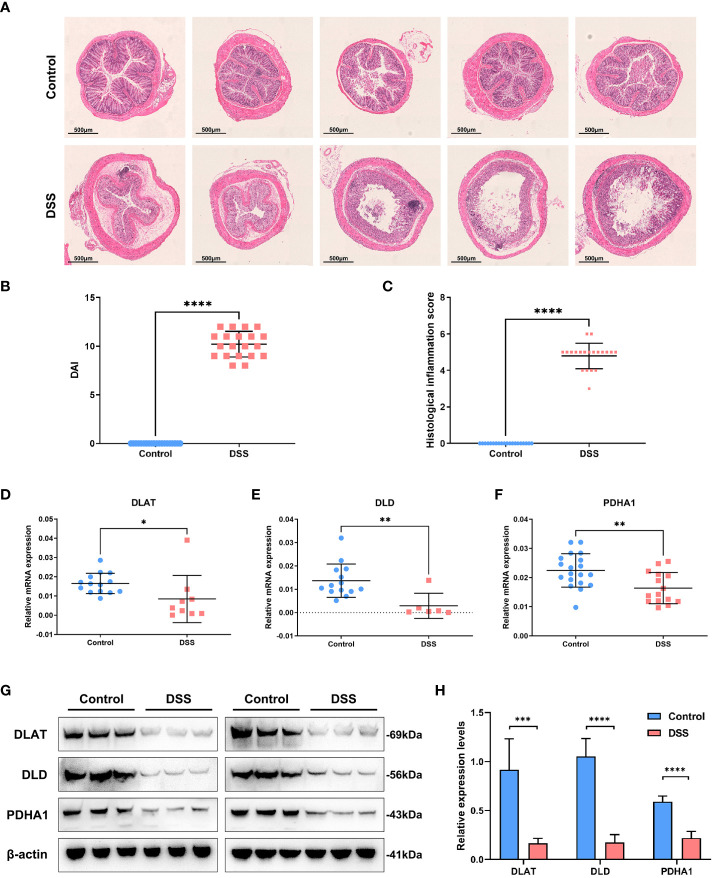
Experimental verification in the UC mouse model. **(A)** Hematoxylin and eosin-stained sections depicting mouse colonic mucosa in the control and DSS groups. **(B)** DAI scores increased after acute administration of DSS. **(C)** Histological inflammation scores increased after DSS treatment. **(D–F)** The expression levels of *DLAT*, *DLD*, and *PDHA1* were detected by using real-time quantitative polymerase chain reaction. **(G, H)**The expression levels of *DLAT*, *DLD*, and *PDHA1* were detected by Western blot analysis in the UC mouse model. (*p < 0.05; **p < 0.01; ***p < 0.001;****p < 0.0001)

The body weight of the mice in each group indicated different changes. We observed that the dextran sulfate sodium (DSS) group indicated a significant and continuous decrease on the 5th day of modeling (*vs*. control, *p* < 0.001) (**Figure 6A**). Notably, colon length is an essential index of the degree of colonic inflammation. Our investigations revealed that colon length in the DSS group was substantially shorter than that in the control group (**Figure 6B**). The mice were sacrificed on the 7th day. The mice in the treatment group weighed less than those in the control group (**Figure 6C**). We found that the colon/body weight was higher in DSS-treated mice than in the control group mice (**Figure 6D**). The DSS-treated mice indicated a reduced colon length (**Figure 6E**). The colon weight/length ratio increased after DSS administration (**Figure 6F**). The weight of the spleen in the DSS group was significantly higher than that in the control group (**Figure 6G**). An increase in the spleen/body weight ratio was also observed after the acute administration of DSS (**Figure 6H**). Hematoxylin and eosin-stained slides indicated that the epithelial cells of the mouse colon mucosa were structured and intact, and goblet cells were abundant, without atrophy, deformation, necrosis, or infiltration of inflammatory cells in the control group. In contrast, typical inflammatory symptoms in the acute phase, disappearance of normal pathological structures, destruction of crypt structures, obvious reduction or even disappearance of goblet cells, and large infiltration of inflammatory cells in the mucosa and submucosa were observed in the DSS group (**Figure 7A**). Furthermore, DAI and histological inflammation scores increased after acute administration of DSS (**Figures 7B, C**). We detected *DLAT*, *DLD*, and *PDHA1* gene expression levels by using real-time quantitative polymerase chain reaction (qRT-PCR) and Western blot analysis (**Figures 7D-H**). Consistent with the results of bioinformatic analysis, *DLAT*, *DLD*, and *PDHA1* were lower than those in the control group. In summary, these three genes play important roles in the development of UC.

## 4 Discussion

Ulcerative colitis is a widespread multicausal inflammatory bowel disease. This is marked by extended clinical courses and recurring bouts. The etiology of this condition is unknown. This is because many factors make interpreting the disease process and diagnosis challenging (33–36).

Chronic inflammation has long been associated with cancer development. Ulcerative colitis is an inflammatory bowel disease that commonly progresses to CRC. Furthermore, CRC is more likely to occur in patients with UC, and the risk is associated with the duration of illness and the range and accumulated burden of inflammation. Moreover, UC causes colonic inflammation that can affect only the rectum or a portion of or the entire colon. The clinical symptoms include diarrhea, gastrointestinal bleeding, and weight loss. Additionally, CRC growth is a serious complication of chronic inflammation (37–39). Therefore, understanding UC pathogenesis and the molecular mechanism of the altered inflammatory environment is vital for UC treatment and CRC caused by UC.

Copper is a typical metal element and transition metal with redox activity. Reduced Cu^+^ can be converted into oxidized Cu^2+^ under standard chemical and physiological conditions. Copper ions play an important role in many biological activities, either by providing or absorbing electrons. Copper ions, as cofactors or structural components, can bind to numerous proteins or enzymes and are involved in a wide range of physiological processes, such as energy metabolism, mitochondrial respiration, and antioxidation (40–43). The dynamic equilibrium that maintains the concentration of Cu ions can result in oxidative stress and aberrant autophagy, which can cause a number of disorders associated with Cu or Cu ions. A new Cu-dependent cell death mode called cuproptosis was proposed in a recent study. It is different from other types of cell death, such as apoptosis, pyroptosis, and necroptosis, but is comparable to ferroptosis in that it is a regulated cell death induced by metal ions (43–45). Furthermore, Cu^2+^ that directly reacts with the fatty acylated portion of the triacyl acid cycle causes fatty acylated proteins to aggregate and makes the iron and sulfur proteins unstable. This causes protein-toxic stress and cell death independent of the cell-flushing process (28, 46).

Recently, researchers found that cuproptosis plays important roles in various human cancers. According to a recent report, cuproptosis-related genes influence the tumor microenvironment in mammary and liver cancers. In addition, cuproptosis is associated with immune infiltration of melanoma and clear cell renal cell carcinoma (43, 47–49). Most of the studies used bioinformatic methods for analyses. Furthermore, recent studies indicate that Cu bioavailability also affects CRC (50). Most UC patients will suffer from CRC. Cuproptosis and the pathophysiology of UC are probably related. Cuproptosis-related genes have also been reported to be widely associated with other diseases, especially digestive disorders. We also analyzed the expression of *DLD*, *DLAT*, and *PDHA1* in GI cancers in the follow-up. It was found that it may also play an important role in GI cancers. Among them, *DLD* and *PDHA1* were differentially expressed in colon cancer, and *DLAT*, *DLD*, and *PDHA1* were differentially expressed in gastric cancer and liver cancer (**Figures S6A–G**). Studies have shown that cuproptosis is closely related to the prognosis of pan-cancers, activation/inhibition of cancer signature pathways, and regulation of the tumor microenvironment (51). Moreover, the prognostic risk model established by cuproptosis-related lncRNAs has been shown in gastric adenocarcinoma (52), hepatocellular carcinoma (53), non-small cell lung cancer (54), and colorectal cancer (55), all of which provide a certain basis for individual prognosis and drug efficacy. Similarly, risk models constructed by cuproptosis-related miRNA also play a role in the prediction and treatment of hepatocellular carcinoma (56).

In this study, we analyzed different expression patterns between the normal and UC tissue samples in three datasets (GSE38713, GSE87473, and GSE92415) using the limma package in R. The MCODE plugin in Cytoscape was used to identify the most meaningful modules in the PPI network, and three cuproptosis-related genes were identified in the top two networks. To determine whether these cuproptosis-related genes affected disease progression in UC patients, we chose GSE87473 to assess the gene expression of cuproptosis-related genes in UC and normal tissues. We found that the levels of cuproptosis-associated genes are highly heterogeneous in normal and UC patients, suggesting that the levels of different cuproptosis-related genes can influence disease progression in UC patients. Combining multiple gene microarray data, we finally selected the three hub genes *DLD*, *PDHA1*, and *DLAT* for the follow-up studies. Cuproptosis patterns were classified using the ConsensusClusterPlus software package of the R software. Using unsupervised clustering, we finally identified three different modification patterns, including 21 cases of CuClusterA, 34 cases of CuClusterB, and 51 cases of CuClusterC. We studied the expression of three cuproptosis-associated genes in different CuClusters. We found that the levels of cuproptosis-associated genes were higher in CuClusterB than in CuClusterA and CuClusterC. Next, we compared the differences in immune cell infiltration among the three clusters using ssGSEA analysis. The results indicated that multiple immune cells, except dendritic cells, were observably enriched in the three CuClusters, indicating that cuproptosis-associated genes can influence the immune microenvironment of UC. To identify the differentially expressed genes in the three CuClusters, we applied the limma package in R. Finally, the differential genes in the three CuClusters were intersected, and 10 differential genes were obtained. After differential analysis of the expression of these 10 genes in GSE87473, we found that the expression of *COLA5A3*, *CHN1*, *H19*, *THBS2*, *SELP*, *KIAA0125*, and *EGFL6* was upregulated in UC tissues compared with that of normal tissues. The expression of *SLC9A2*, *FAM55A*, and *ANXA13* was significantly downregulated. These 10 genes exhibited a significant relationship with cuproptosis-related genes, which suggests an underlying role of cuproptosis in UC. We further explored the effect of cuproptosis phenotype-associated hub genes on the immune infiltration of UC. Cuproptosis-related hub gene expression possibly affects the efficacy of infliximab, indicating that the cuproptosis phenotype can affect immunotherapy. These results suggest that the high expression of some hub genes associated with abnormal epidermal phenotypes in UC patients can be associated with resistance to infliximab. Drug selection and the response to anti-infliximab therapy in UC patients can be determined by assessing the expression of cuproptosis phenotype-associated hub genes. We performed experimental verification to test our bioinformatic analysis. Verification in the UC mouse model revealed that the levels of *DLAT*, *DLD*, and *PDHA1* genes of the DSS group were observably lower than those of the control group, which was consistent with the results of bioinformatic analysis. *DLD* is one of the components of the lipoic acid pathway, and *DLAT* and *PDHA1* belong to the pyruvate dehydrogenase complex. The downregulation of the three genes prevents cuproptosis, which is consistent with our experimental results. These results indicate that *DLAT*, *DLD*, and *PDHA1* have important functions in the development of UC.

## 5 Conclusion

This is the first study to identify cuproptosis subtypes and develop a predictive model for UC (**Figure S7**). Cuproptosis, which varies from the other documented ways of cell death, can facilitate new treatment avenues for UC. Furthermore, a number of approaches and databases were used. We also identified the cuproptosis subtypes and developed a predictive model. This model improves the reliability of our findings. In this study, we carefully examined how the immunological microenvironment and cuproptosis-related genes are linked to the prognosis of UC. In the UC mouse model, we also investigated the efficiency of cuproptosis hub genes. As a result, our work highlights the significance of cuproptosis and provides a solid foundation for future studies on the identification of individualized care for UC patients.

## Data availability statement

Publicly available datasets were analyzed in this study. These data can be found here: https://www.ncbi.nlm.nih.gov/geo/.

## Ethics statement

The animal study was reviewed and approved by The Inspection of the Institutional Animal Care and Use Committee of The Third Xiangya Hospital, Central South University.

## Author contributions

WW and ZY designed the study and wrote the manuscript. CY and SL performed the majority of the experiments. WDW and ZQ performed most of the bioinformatic analyses. XM, MY, JZ, LC and SY wrote the manuscript. All authors contributed to the article and approved the submitted version.
