# Supplementary material for: Identification of cuproptosis hub genes contributing to the immune microenvironment in ulcerative colitis using bioinformatic analysis and experimental verification
Source: Front Immunol. 2023 Mar 7;14:1113385. doi: 10.3389/fimmu.2023.1113385 (PMC10028083; doi:10.3389/fimmu.2023.1113385)
Supplement: Supplementary file 1 [file DataSheet_1.docx]

Supplementary Material

Bioinformatics Analysis and Experimental Verification Identify Cuproptosis Hub Genes Contributing to Immune Microenvironment in Ulcerative colitis to Prevent Associated Colorectal Cancer

**Cejun Yang*, Wendi Wang, Sang Li, Zhengkang Qiao, Xiaoqian Ma, Min Yang, Juan Zhang, Lu Cao, Shanhu Yao, Zhe Yang* and Wei Wang***

*** Correspondence:** Corresponding Author: Wei Wang, [wang_w@csu.edu.cn](mailto:wang_w@csu.edu.cn);

Zhe Yang, [yangzhe@lnu.edu.cn](mailto:yangzhe@lnu.edu.cn)

**Supplementary Figures**

**
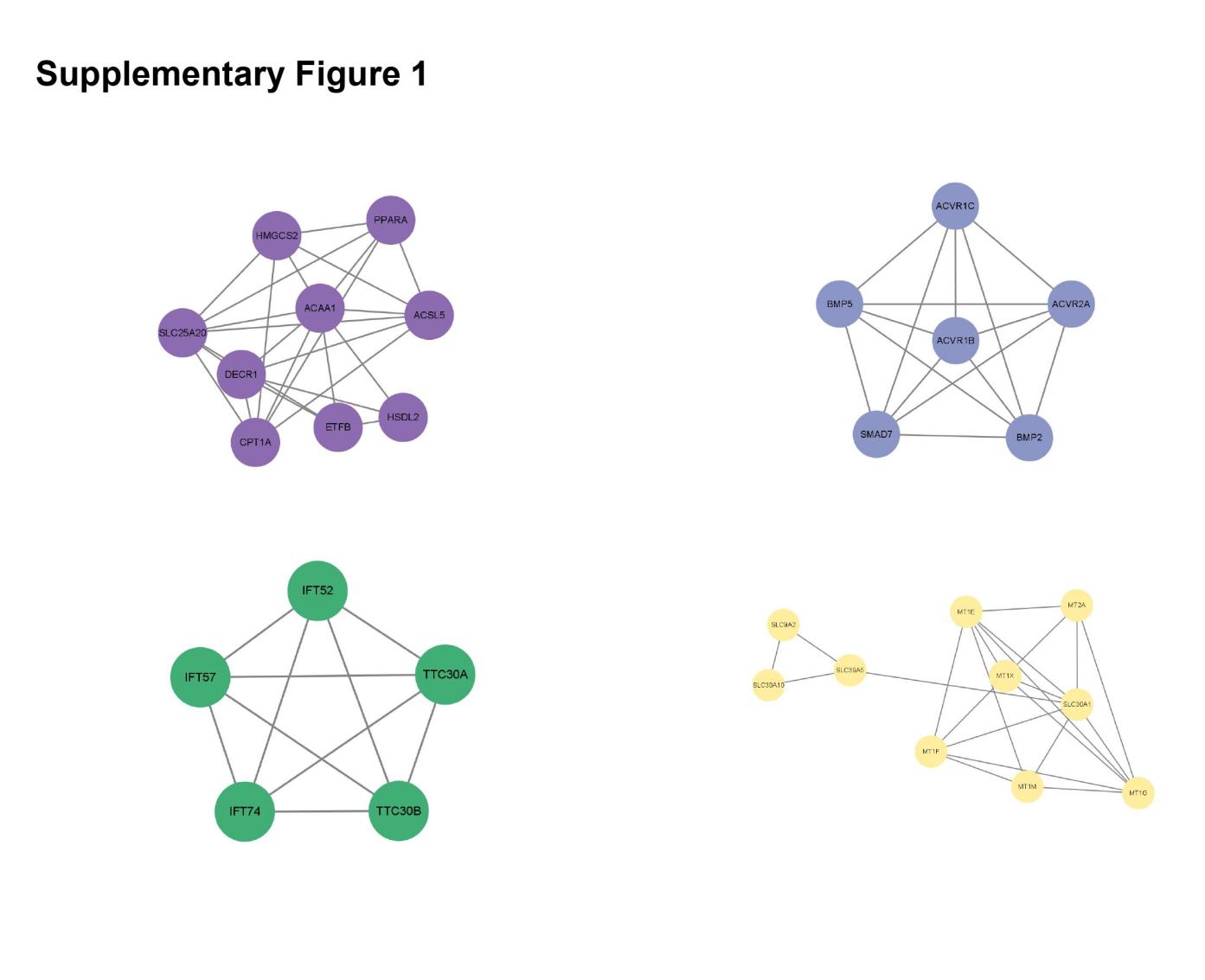
**

**Supplementary Figure 1.** Use the MCODE plugin in Cytoscape to identify the most important modules in a PPI network


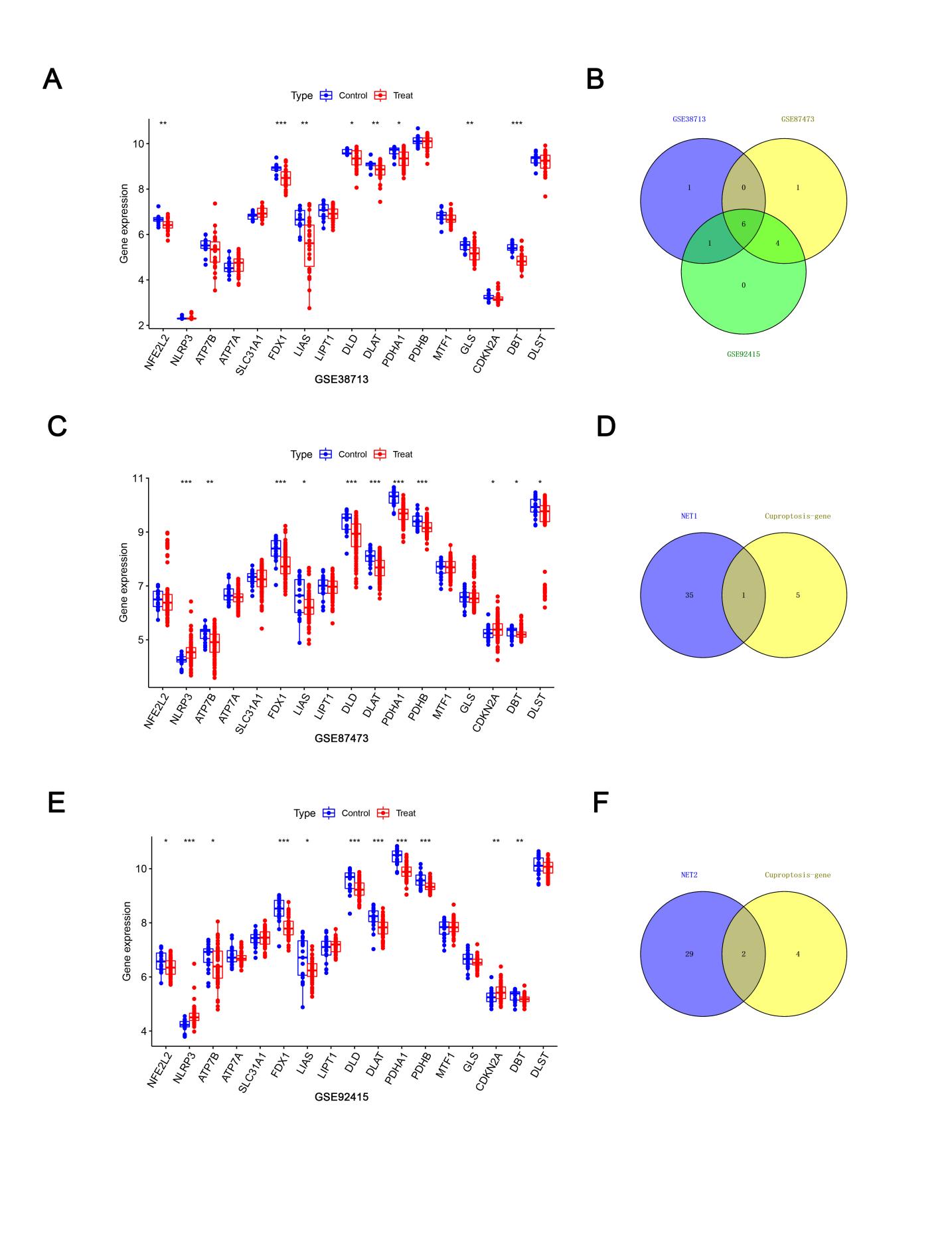


**Supplementary Figure 2. Screening process of 3 cuproptosis-related genes**

(A) Expression of cuproptosis-related genes in GSE38713. (B) The number of intersecting differential cuproptosis-related genes between GSE38713, GSE92415, GSE87473. (C) Expression of cuproptosis-related genes in GSE87473. (D) Venn diagram showing the overlap between the differentially expressed cuproptosis-related genes and the genes contained in the sub-network NET1. (E) Expression of cuproptosis-related genes in GSE92415. (F) Venn diagram showing the overlap between the differentially expressed cuproptosis-related genes and the genes contained in the sub-network NET2.


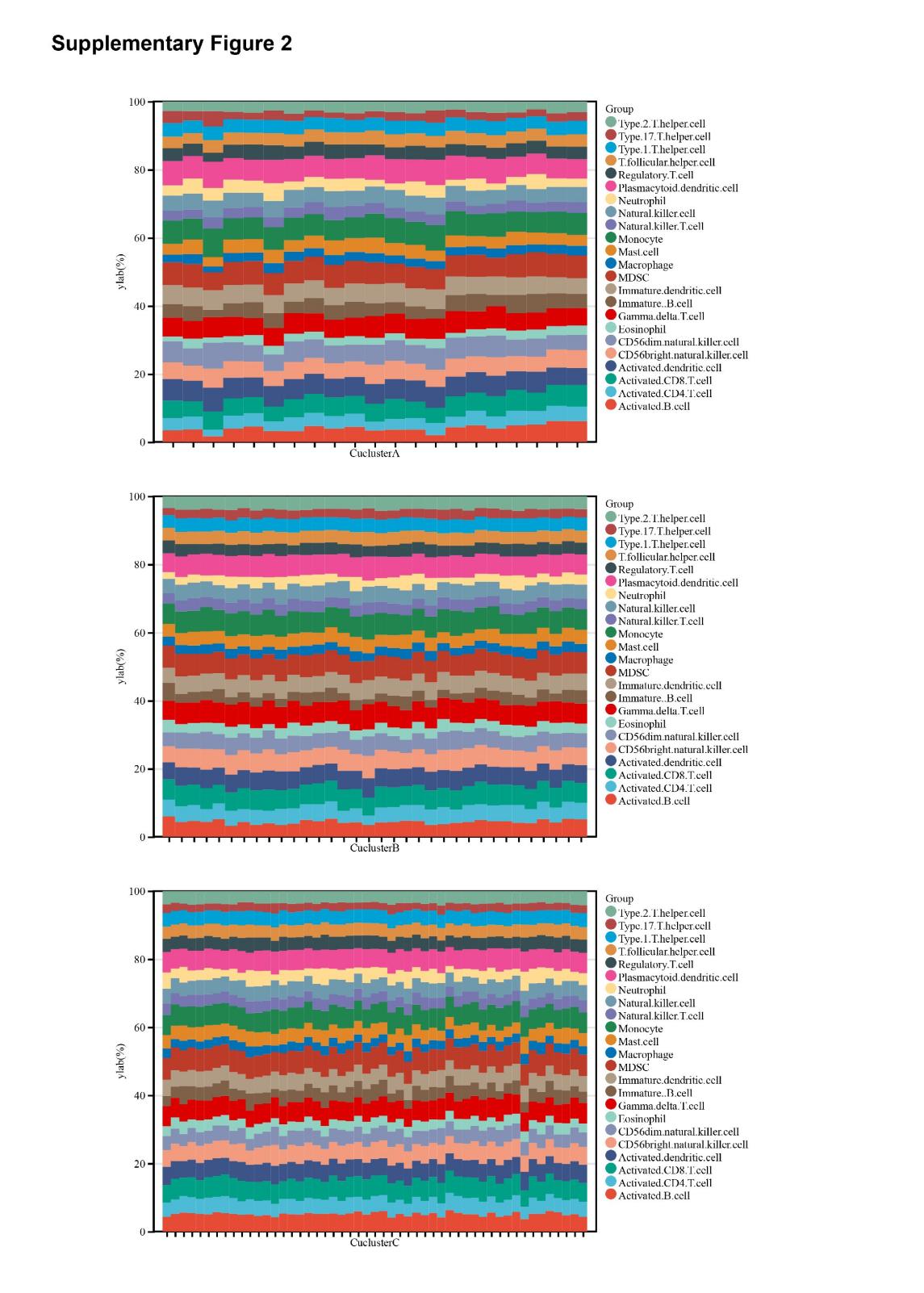


**Supplementary Figure 3.** The relationship between each CuCluster subtype and the level of immune cell infiltration


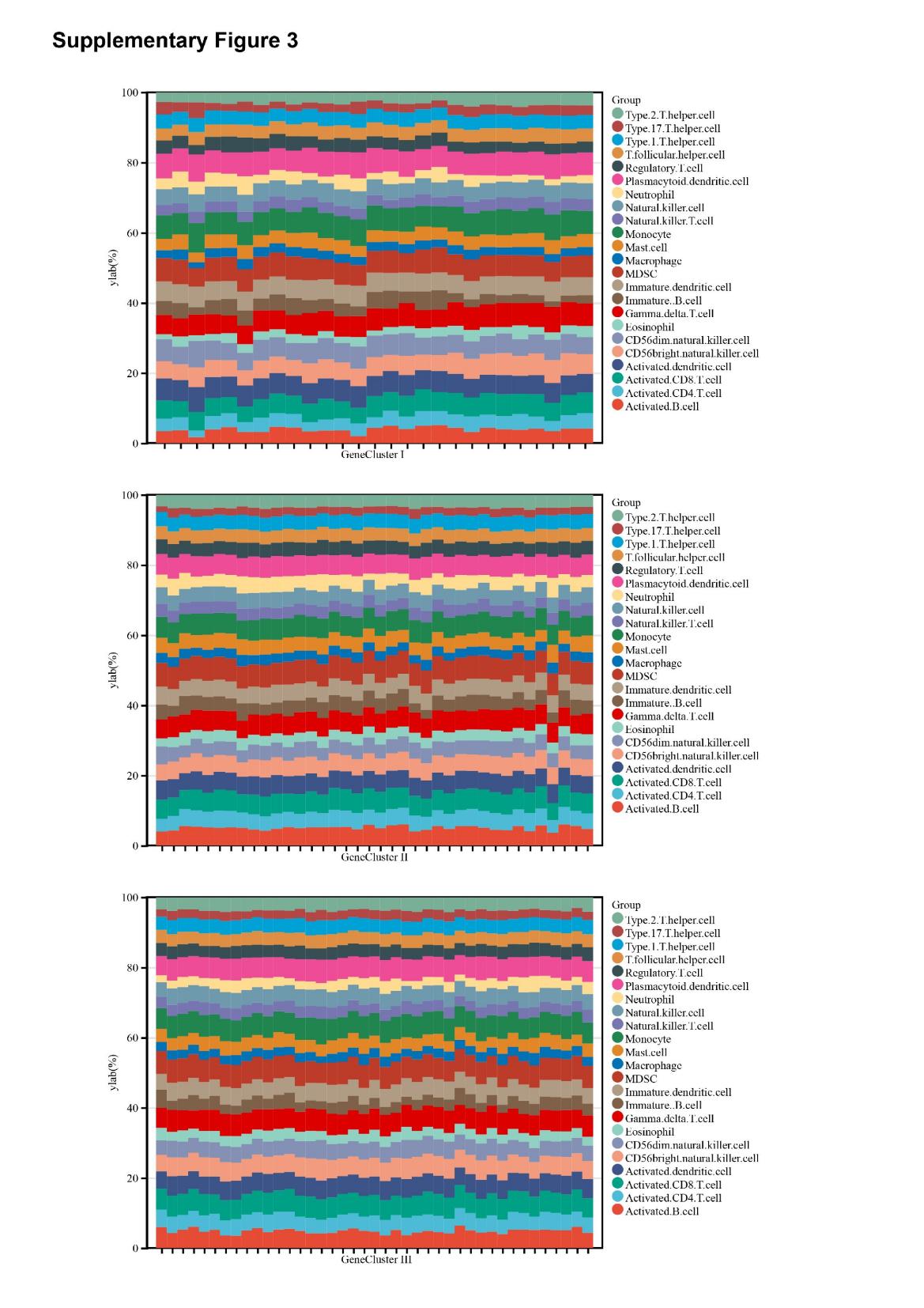


**Supplementary Figure 4.** The relationship between each GeneCluster subtype and the level of immune cell infiltration


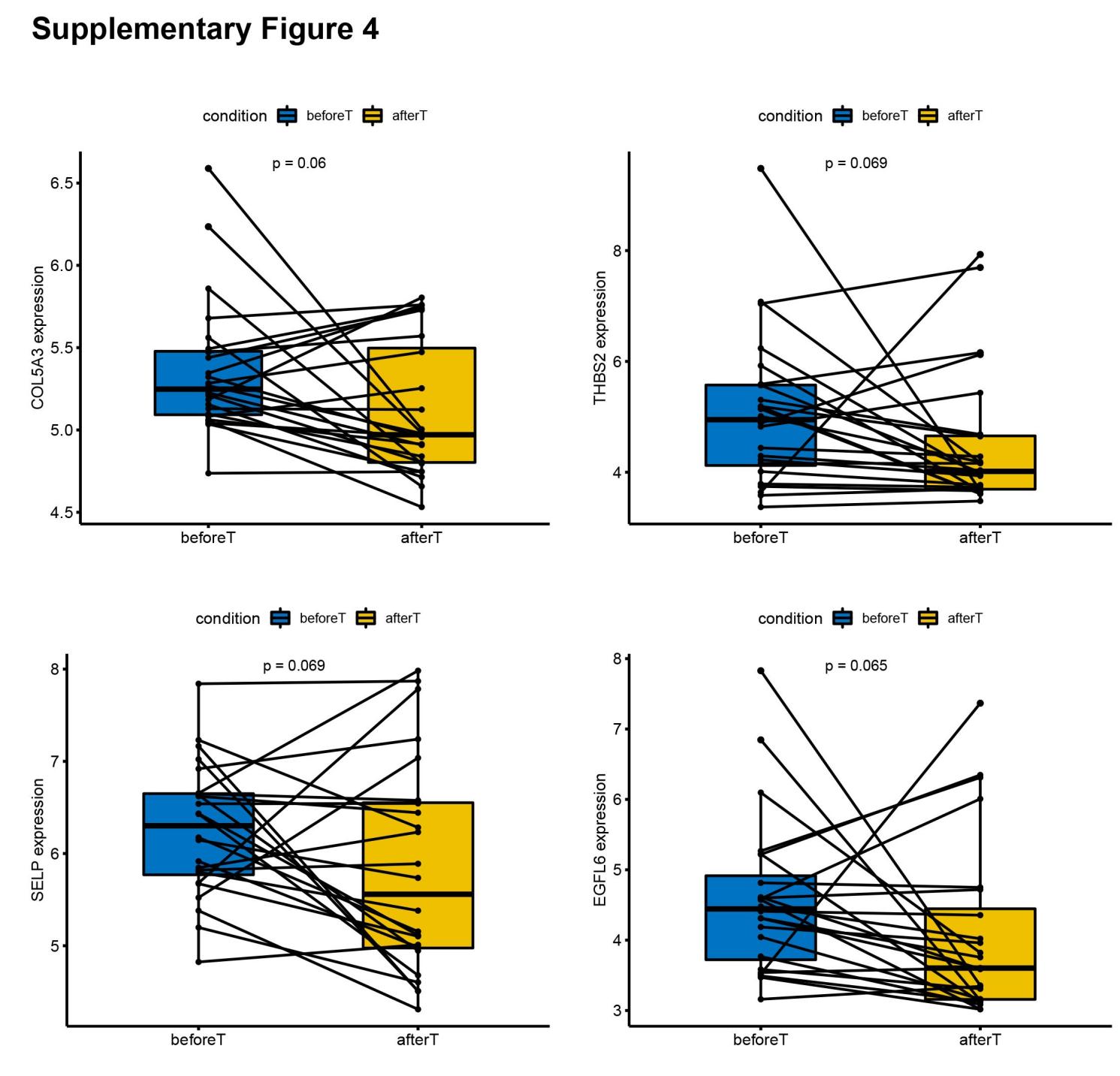


**Supplementary Figure 5.** Hub genes correlations were analyzed in responders before and after infliximab treatment.


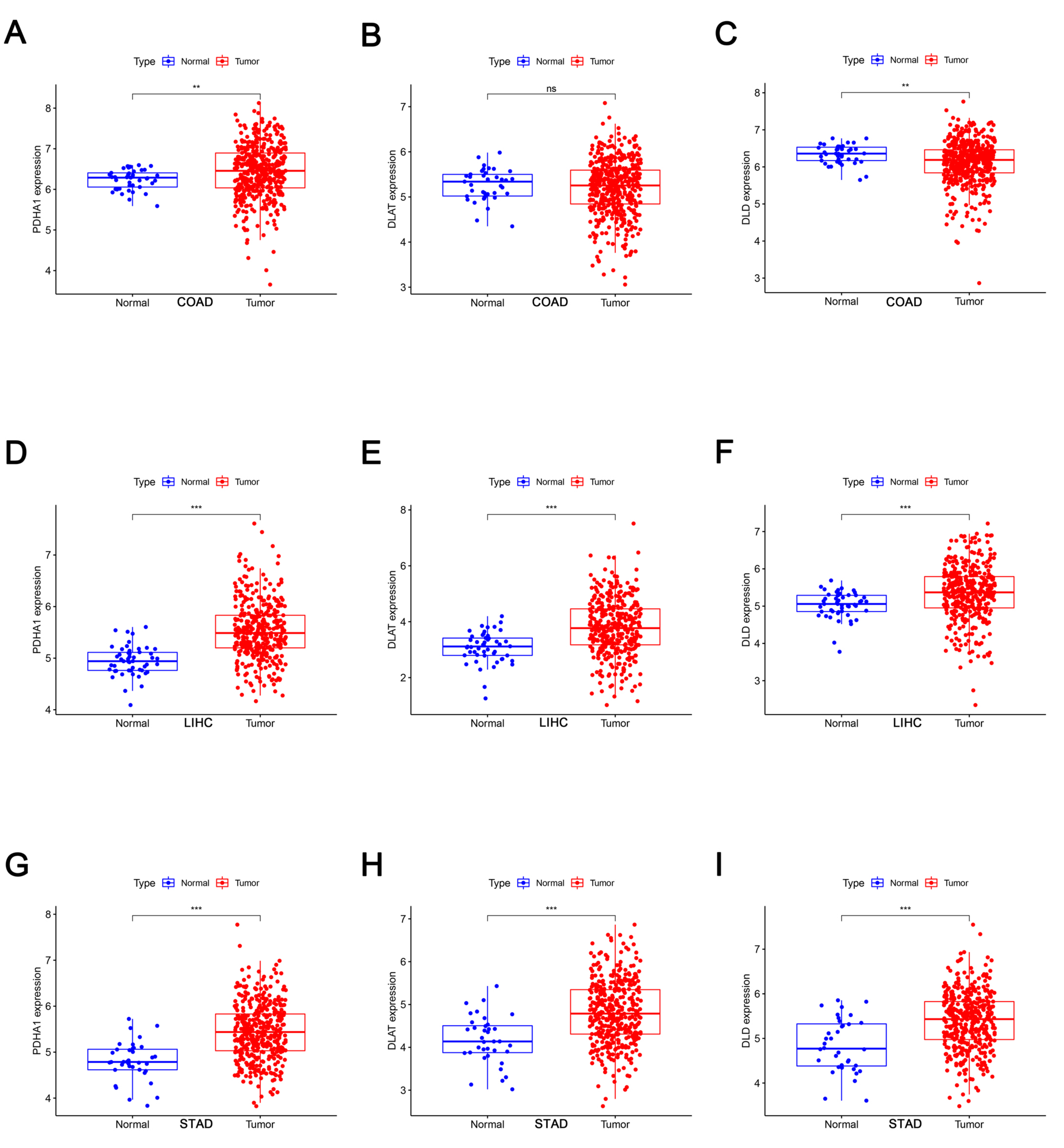


**Supplementary Figure 6. Differential expression of PDHA1, DLAT, and DLD in colon, gastric, and hepatocellular carcinomas.**

(A) Differential expression of PDHA1 in COAD. (B) Differential expression of DLAT in COAD. (C) Differential expression of DLD in COAD. (D) Differential expression of PDHA1 in LIHC. (E) Differential expression of DLAT in LIHC. (F) Differential expression of DLD in LIHC. (G) Differential expression of PDHA1 in STAD. (H) Differential expression of DLAT in LIHC. (I) Differential expression of DLD in LIHC.


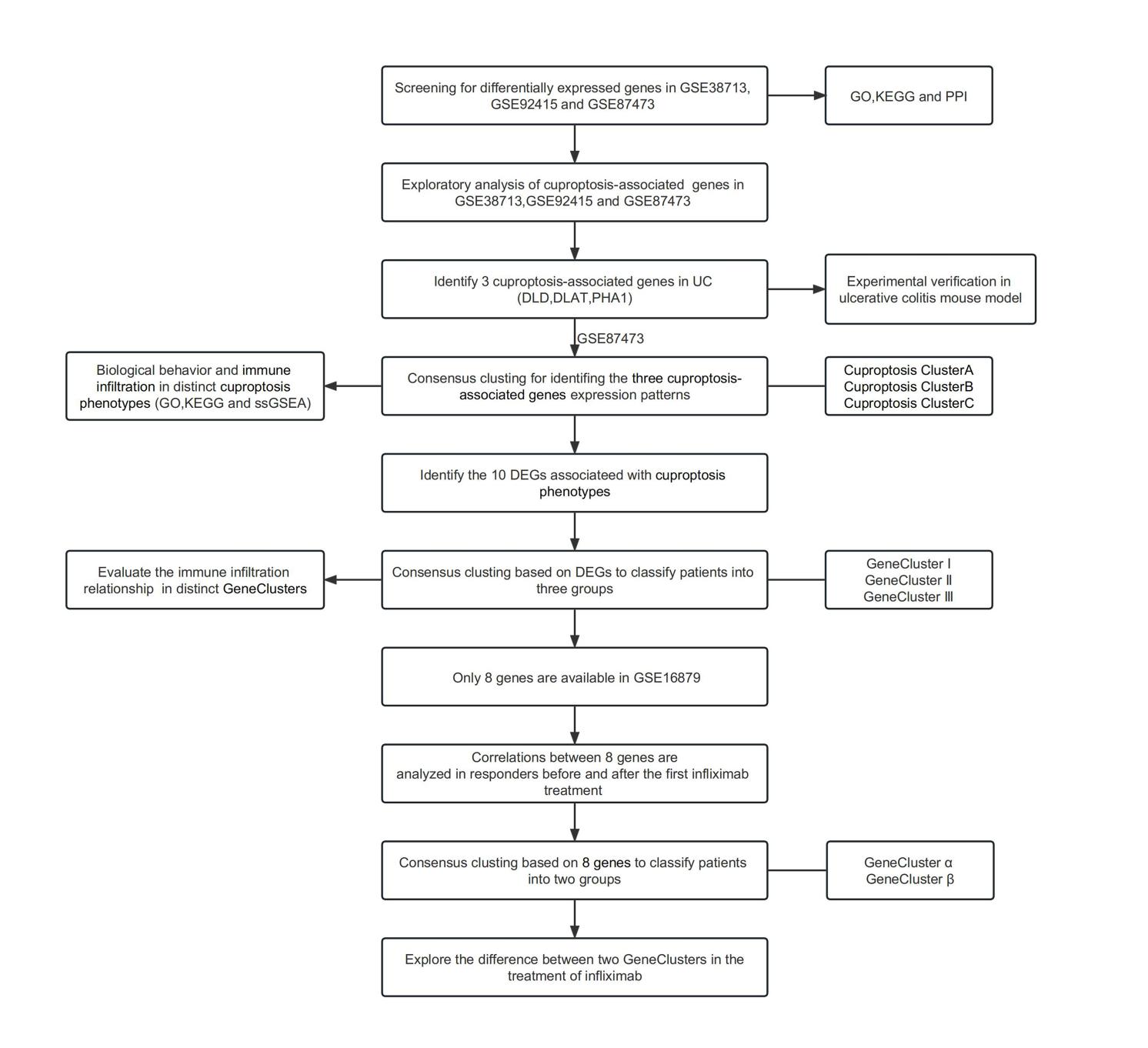


**Supplementary Figure 7. The flowchart of the entire study.**
